# Cytotoxic and targeted therapy for treatment of pseudomyogenic hemangioendothelioma

**DOI:** 10.1186/s13569-015-0037-8

**Published:** 2015-10-19

**Authors:** Jason Joseph, Wei-lien Wang, Madhavi Patnana, Naveen Ramesh, Robert Benjamin, Shreyaskumar Patel, Vinod Ravi

**Affiliations:** Department of Sarcoma Medical Oncology, Unit 0450, The University of Texas MD Anderson Cancer Center, 1515 Holcombe Blvd., Houston, TX 77030 USA; Department of Pathology, The University of Texas MD Anderson Cancer Center, Houston, TX USA; Department of Diagnostic Radiology, The University of Texas MD Anderson Cancer Center, Houston, TX USA

**Keywords:** Pseudomyogenic, Hemangioendothelioma, Soft tissue tumor, Sarcoma, mTOR inhibitor

## Abstract

Pseudomyogenic hemangioendothelioma (PMH) is a recently described, indolent vascular tumor that usually presents in the distal extremities. PMH typically has a multi-focal presentation and can involve several tissue planes including the dermis, subcutis, muscle, and bone. This soft tissue tumor predominantly affects men between 20 and 50 years of age. PMH tumors typically are resected but frequently recur locally; thus, more efficacious treatment options are needed. Herein, we report two cases of patients with PMH who were treated with systemic therapy. To the best of our knowledge, our report is the first to describe a response of PMH either to gemcitabine/taxane cytotoxic chemotherapy or to a mammalian target of rapamycin inhibitor. In the first case, a 45-year-old man with PMH of the right ilium was treated with gemcitabine plus docetaxel. Although chemotherapy was ultimately halted owing to gemcitabine-induced pulmonary toxicity, positron emission tomography-computer tomography scans taken after three cycles of gemcitabine plus docetaxel illustrated a noticeable response to the regimen. In the second case, a 22-year-old man with PMH of the right distal femur and metastases in the left ilium showed no response to gemcitabine plus docetaxel therapy, but underwent surgical resection after cisplatin and doxorubicin resulted in stable disease. DNA sequencing of his tumor revealed the presence of a tuberous sclerosis 1 (TSC1) mutation, so daily everolimus, which inhibits mammalian target of rapamycin, was started. Two months after beginning everolimus, the patient underwent magnetic resonance imaging of the pelvis, which revealed mild shrinkage of PMH metastases in the left iliac bone. Despite the apparent heterogeneity of response to gemcitabine/taxane chemotherapy in our two patients, these two cases indicate that gemcitabine/taxane and mammalian target of rapamycin inhibitor may serve as systemic treatment options for PMH and warrant further investigation.

## Background

Pseudomyogenic hemangioendothelioma (PMH) is a rare indolent vascular tumor that typically presents in the distal extremities and may present in multiple tissue planes, including the dermis, subcutis, muscle, and bone [1, 2]. Over the last three decades, PMH has been determined to be the same pathological entity as epithelioid sarcoma-like hemangioendothelioma and fibroma-like variant of epithelioid sarcoma [2–5]. The tumor has a 4.6:1 male predominance and typically occurs in men between 20 and 50 years of age. Histopathologically, PMH resembles a myogenic neoplasm with a striking rhabdomyoblast appearance and spindle cell morphology. However, PMH lacks true muscle markers such as desmin and has an immunophenotype and highly membranous pattern in line with endothelial differentiation [2, 5]. PMH has recently been characterized molecularly to have a balanced t(7;19) translocation resulting in a SERPINE1-FOSB fusion [6]. PMH does not typically transform into high-grade disease or metastasize. However, PMH frequently recurs locally after excision [2].

While much has been published concerning the characterization of PMH, much remains to be learned about the natural history of the disease and efficacy of treatment. In the majority of PMH cases, excision is the management modality of choice, but over a third of patients experience local recurrence or new nodules in adjacent soft tissue during follow up [2]. Previous accounts demonstrate an indolent course of disease and most studies have shown that the disease does not progress before or after therapy. In a large case series (n = 50), only one patient died of PMH. Nevertheless, since PMH presents as multifocal tumors in 70 % of patients, systemic therapy options are acutely needed [2].

To the best of our knowledge, only two published reports [5, 7] in the literature discuss a response by PMH to therapeutic options other than excision.

Here, we report two cases of patients who had PMH that responded to systemic therapy: one patient exhibited a noticeable response to gemcitabine plus docetaxel and one patient demonstrated a response to everolimus, a mammalian target of rapamycin (mTOR) inhibitor.

## Case presentation

### Case 1

In October 2010, a 45-year-old man presented to his local physician with pain in his right groin, which the patient attributed to a muscle strain from playing soccer. As his pain worsened, the patient went to an orthopedic surgeon who obtained a magnetic resonance imaging (MRI) study that showed a mass within the right anterior ilium. In December 2010, the patient was examined by physicians in the Department of Orthopaedic Oncology at The University of Texas MD Anderson Cancer Center, and a biopsy of the right ilium mass revealed a malignant epithelioid and spindle cell neoplasm most consistent with PMH. The patient was treated with two cycles of intra-arterial cisplatin (120 mg/m^2^) administered every 3 weeks. Due to hearing loss, administration was modified to intravenously with a slow infusion rate over 24 h for the next two cycles. Restaging positron emission tomography-computed tomography (PET-CT) studies revealed a minor tumor response to cisplatin.

**Fig. 1:**
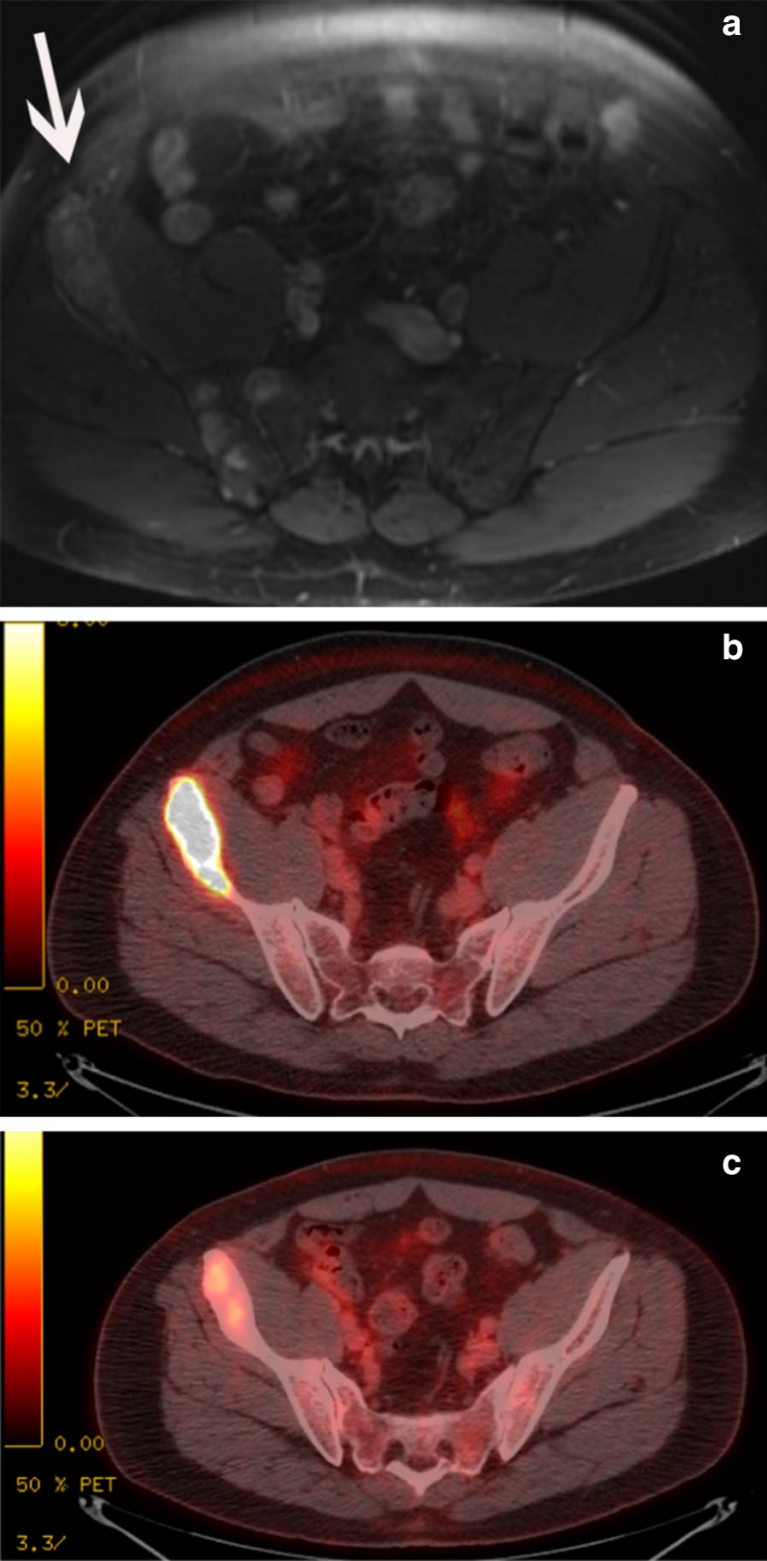
Imaging studies for 45-year-old patient (“Case 1”) with pseudomyogenic hemangioendothelioma. **a** Baseline fat saturated T1-weighted axial magnetic resonance image with intravenous contrast enhancement of the pelvis demonstrates multiple enhancing tumor foci in the right iliac bone (*white arrow*). **b** Positron emission tomography-computed tomography (PET-CT) fusion image through the pelvis demonstrates diffuse metabolic activity in the right anterior iliac bone (disease in the right posterior iliac bone at the level of the sacroiliac joint was also present but is not shown). This image was obtained 1 month before the patient began gemcitabine/docetaxel. **c** PET-CT fusion image through the pelvis after three cycles of gemcitabine/docetaxel demonstrates a significant metabolic response

Because the patient’s hearing loss worsened, therapy was switched to a combination of gemcitabine (900 mg/m^2^) and docetaxel (100 mg/m^2^) administered in 3 week cycles, beginning 3 weeks after cessation of cisplatin. Restaging PET-CT studies performed after completion of the third cycle revealed that the PMH had significantly responded to gemcitabine plus docetaxel (Fig. 1). Unfortunately, despite this significant improvement, there was evidence of gemcitabine-induced pulmonary toxicity. Therefore, cytotoxic chemotherapy was halted, and a tapering dose of steroids was given to the patient to treat the pulmonary toxicity. The patient’s edema was managed with furosemide and amiloride.

During a follow-up visit 3 months after cessation of gemcitabine and docetaxel, the patient’s PET-CT scans showed mild progression of disease in the right iliac bone with enlarging lucencies. This finding suggested that the PMH progressed in the absence of gemcitabine and docetaxel treatment. Therapy with weekly paclitaxel (80 mg/m^2^) was therefore started. However, after 4 months of paclitaxel, therapy was discontinued because the patient experienced hearing loss, rhinitis, and neuropathy characterized by tingling of the hands and feet. Restaging studies performed 16 months following cessation of cytotoxic chemotherapy revealed stable disease.

The tumor response to the combination of gemcitabine and a taxane was clearly demonstrated on the PET-CT scans. PMH progressed once chemotherapy was stopped, and taxane treatment led to disease stability, suggesting that gemcitabine plus taxane could be an effective treatment for PMH.

### Case 2

**Fig. 2:**
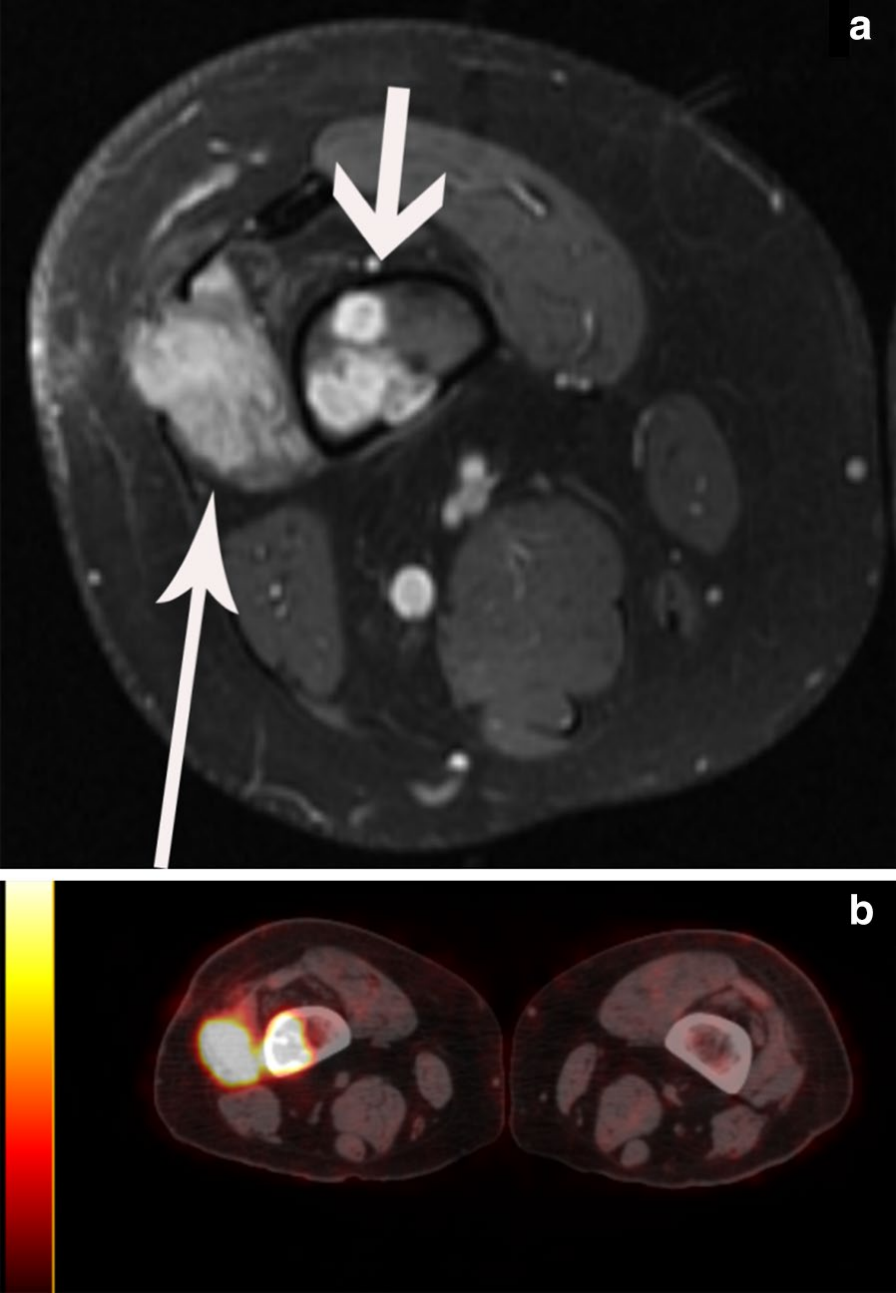
Baseline magnetic resonance imaging (MRI) and positron emission tomography-computed tomography (PET-CT) studies for a 22-year-old man (“Case 2”) with pseudomyogenic hemangioendothelioma. **a** Baseline contrast-enhanced, fat-saturated T1-weighted axial MR image of the right thigh demonstrates multifocal enhancing lesions in the distal femoral metadiaphysis (*short white arrow*) and vastus intermedius muscle and subcutaneous fat. **b** PET-CT fusion image through the distal femurs demonstrates corresponding hypermetabolic activity. Restaging examinations after two cycles of gemcitabine and docetaxel (not shown) did not demonstrate any significant response

In June 2011, a 22-year-old man presented to his local physician with a painless, dime-shaped nodule in his right lateral thigh. Approximately 1 year later, the mass began to enlarge and induce pain, and the patient saw a dermatologist who performed a punch biopsy that was inconclusive. In September 2012, an excisional biopsy was performed at MD Anderson Cancer Center, and the pathologist concluded that the mass was consistent with PMH originating from the right femur. Baseline imaging included MRI of the right thigh which showed multiple enhancing lesions in the distal right femur and lateral soft tissues, extending for a length of 6.8 cm and centered approximately 7 cm proximal to the articular surface of the lateral femoral condyle. A baseline PET-CT study revealed a hypermetabolic mass in the right distal femur (standardized uptake value, maximum of 15.2) and a 1.3 cm left acetabular lytic lesion (standardized uptake value, maximum of 7.9), suspicious for metastatic disease (Fig. 2). After the patient was evaluated by physicians in the Department of Sarcoma Medical Oncology at MD Anderson, therapy with gemcitabine (900 mg/m^2^) and docetaxel (75 mg/m^2^) was started and given in 3 week cycles. The cytotoxic chemotherapy was well tolerated. However, imaging showed no evidence of tumor response after two cycles, and treatment was halted.

Therapy was then changed to doxorubicin (90 mg/m^2^ as a continuous infusion over 72 h) and cisplatin (120 mg/m^2^ as an intravenous infusion over 4 h), but the patient had mucositis, tinnitus, neutropenic fever, and ototoxicity during the first cycle. The cisplatin dose was reduced to 100 mg/m^2^ as a slow infusion over 24 h to prevent ototoxicity and doxorubicin was changed to bolus doxorubicin with dexrazoxane (90 mg/m^2^) in a 3 week cycle to reduce mucositis caused by continuous intravenous infusion of doxorubicin. The patient tolerated the regimen better during the second cycle, and the PMH remained stable.

**Fig. 3:**
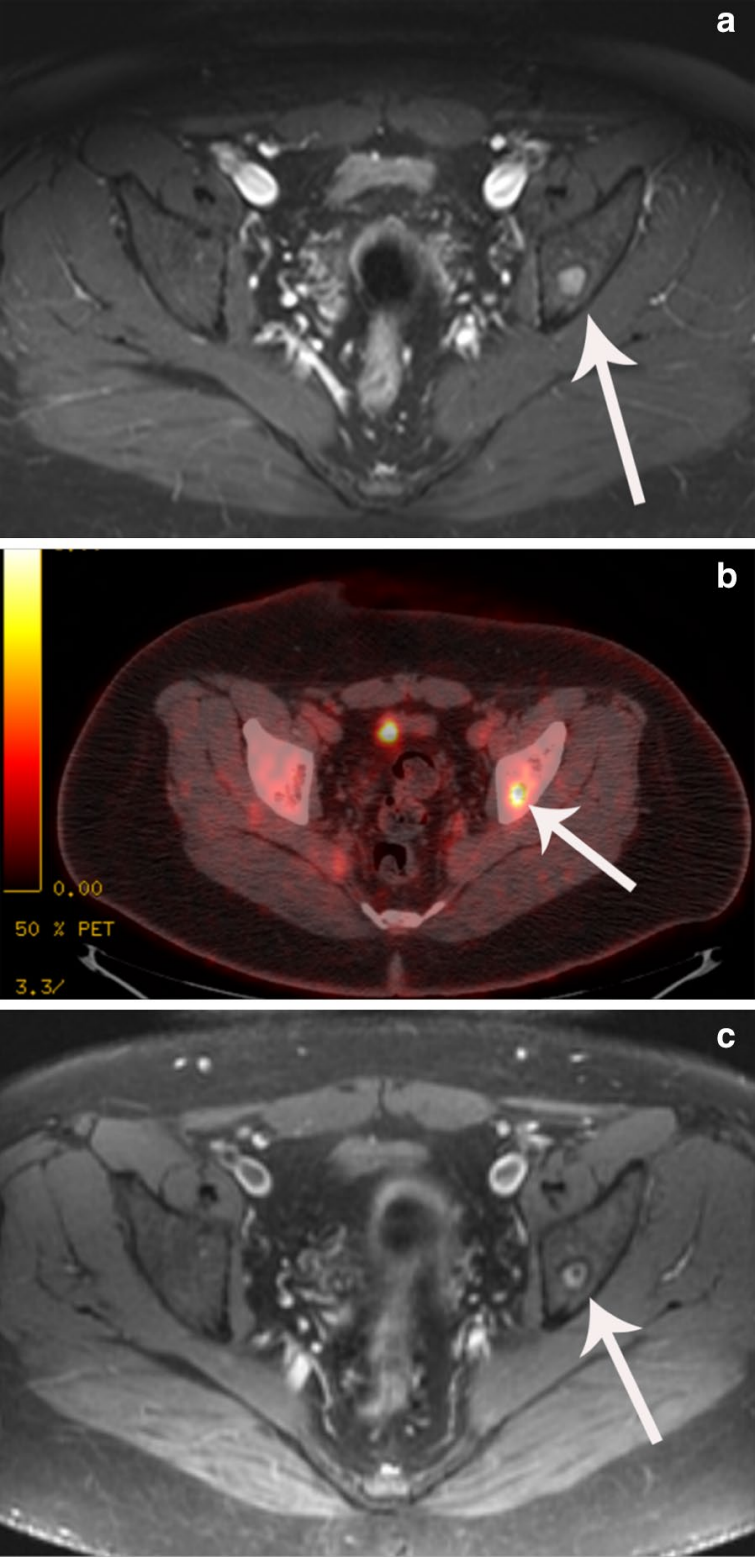
Imaging studies obtained during treatment for a 22-year-old man (“Case 2”) with pseudomyogenic hemangioendothelioma. **a** Fat-saturated T1-weighted axial magnetic resonance image with intravenous contrast enhancement of the pelvis demonstrates a solitary enhancing tumor focus in the supra-acetabular region of the left iliac bone (*white arrow*), before everolimus treatment. **b** Positron emission tomography-computed tomography fusion image through the pelvis demonstrates corresponding metabolic activity. This image was obtained before the patient began everolimus treatment. **c** Fat-saturated T1-weighted axial MR image with intravenous contrast enhancement of the pelvis obtained after 2 months of everolimus demonstrates partial response of the solitary enhancing tumor focus in the supra-acetabular region of the left iliac bone

In July 2013, the patient underwent surgical resection to remove the right distal femoral lesion. DNA sequencing of the tumor revealed the presence of a tuberous sclerosis 1 (TSC1):c.1760A >Gp.K587R mutation. Owing to the TSC1 mutation, therapy with everolimus (10 mg/day) was started. Two months later, the patient underwent MRI of the pelvis, which revealed mild interval healing of the small intramedullary metastases in the supra-acetabular region of the left iliac bone (Fig. 3).

Restaging MRI studies of the pelvis were performed 5 months after the start of everolimus treatment and revealed generally stable disease and no evidence of recurrent disease in the thigh. Owing to the stability of the disease, the patient decided to cease everolimus therapy and pursue cryotherapy. Overall, the mTOR inhibitor provided a noticeable response in the tumor, which had not responded to doxorubicin and cisplatin. Thus, targeted therapy could serve as a viable treatment option for PMH.

## Discussion

To the best of our knowledge, only two previous descriptions of outcomes from PMH therapy other than resection have been published [5, 7]. The first report described a 36-year-old man with multiple lesions in his right lower leg treated with isolated limb perfusion (melphalan and tumor necrosis factor alpha) followed by four cycles of ifosfamide and doxorubicin. The treatment did not elicit significant shrinkage of the tumor, but the lesion remained stable after 9 months of follow-up [5]. The second study reported a response of PMH to metronomic oral cyclophosphamide and prednisolone. The patient’s lesion decreased in size after 10 months of therapy before the patient became non-compliant; however, the report did not include any quantifiable data or imaging concerning lesion size and progression [7].

A review of all published literature on pseudomyogenic hemangioendothelioma was performed to summarize major findings of this newly characterized soft tissue tumor. Of the 63 total reported cases, 78 % of patients were males. PMH presented in the extremities in 79 % of cases and was multifocal at diagnosis in over two-thirds of patients. 90 % of cases utilized excision as the treatment modality of choice (Table 1).

**Table 1:** Reported cases of pseudomyogenic hemangioendothelioma

References	Cases (N)	Sex	Tumor location	Multifocality at diagnosis	Management
Sheng [1]	1	Male: 100 % (1of 1) Female: 0 % (0 of 1)	Extremity: 100 % (1 of 1)	100 % (1 of 1)	Excision: 100 % (1 of 1)
Hornick and Fletcher [2]	50	Male: 82 % (41 of 50) Female: 18 % (9 of 50)	Extremity: 78 % (39 of 50) Trunk: 18 % (9 of 50) Head and neck: 4 % (2 of 50)	66 % (33 of 50)	Excision: 92 % (46 of 50) Post-operative radiation: 16 % (8 of 50) Chemotherapy: 12 % (6 of 50)
Amary et al. [5]	5 (1 case included in Hornick was excluded)^a^	Male: 50 % (2 of 4) Female: 50 % (2 of 4)	Extremity: 100 % (4 of 4)	75 % (3 of 4)	Excision: 75 % (3of 4) Chemotherapy: 25 % (1 of 4)
Stuart et al. [7]	1	Male: 100 % (1 of 1) Female: 0 % (0 of 1)	Extremity: 100 % (1 of 1)	100 % (1 of 1)	Chemotherapy: 100 % (1 of 1)
Sheng and Wang [9]	1	Male: 0 % (0 of 1) Female: 100 % (1 of 1)	Extremity: 100 % (1 of 1)	100 % (1 of 1)	Excision: 100 % (1 of 1)
Requena et al. [10]	2	Male: 50 % (1 of 2) Female: 50 % (1 of 2)	Extremity: 50 % (1 of 2) Head and neck: 50 % (1 of 2)	100 % (2 of 2)	Excision: 100 % (2 of 2)
Righi [11]	2	Male: 50 % (1 of 2) Female: 50 % (1 of 2)	Extremity: 100 % (2 of 2)	100 % (2 of 2)	Excision: 100 % (2 of 2)
Karakasli et al. [12]	1	Male: 100 % (1 of 1)	Extremity: 100 % (1 of 1)	0 % (0 of 1)	Excision: 100 % (1 of 1)
McGinity [13]	1	Male: 100 % (1 of 1) Female: 0 % (0 of 1)	Extremity: 0 % (0 of 1) Trunk: 100 % (1 of 1)	0 % (0 of 1)	Excision: 100 % (1 of 1)
Total	63	Male: 78 % (49 of 63) Female: 22 % (14 of 63)	Extremity: 79 % (50 of 63) Trunk: 16 % (10 of 63) Head and neck: 5 % (3 of 63)	Multifocality at diagnosis: 68 % (43 of 63)	Excision: 90 % (57 of 63) Post-op radiation: 13 % (8 of 63) Chemotherapy: 13 % (8 of 63)

^a^Patient 5 of this report was already accounted for in the series by Hornick et al. [2]

In the first of our cases, the patient exhibited a noticeable treatment response to gemcitabine plus docetaxel. As the 3 month follow-up PET-CT scans clearly indicate, a significant tumor response was achieved following therapy (Fig. 1). The progression of the tumor 3 months after cessation of chemotherapy suggests that the tumor response directly resulted from the gemcitabine and docetaxel therapy. Furthermore, the resumption of a taxane led to disease stability, suggesting that gemcitabine plus a taxane could be an effective treatment for PMH.

Unlike patient 1, patient 2’s tumor did not respond to gemcitabine plus docetaxel but ultimately showed stability following treatment with cisplatin and doxorubicin. The differing outcomes following gemcitabine plus docetaxel treatment in the two cases reflect PMH’s heterogeneity of response to cytotoxic chemotherapy. The discovery of a TSC1 mutation in patient 2’s tumor prompted the use of an mTOR inhibitor. An mTOR inhibitor was selected due to its potential to inhibit aberrant mTOR signaling in perivascular epithelioid cell tumors with similar TSC1 mutations [8]. The mTOR inhibitor used in case 2 provided a noticeable response in the PMH metastases in the supra-acetabular region of the left iliac bone. Thus, systemic therapy targeted to the mutations detected by DNA sequencing could be a promising option for PMH that warrants further investigation.

## Conclusion

To the best of our knowledge, our report is the first to describe a response of PMH either to gemcitabine/taxane chemotherapy or to an mTOR inhibitor. These observations are especially noteworthy because previous treatment options have typically dealt with local excision and a high rate of recurrence or chemotherapeutic approaches which lack a significant response. There is much to be learned about systemic treatment of pseudomyogenic hemagioendothelioma, but these two cases demonstrate two distinct treatment approaches that warrant further investigation.

## Consent

Written informed consent was obtained from the patients for publication of this case report and any accompanying images. A copy of the written consent is available for review.

## Abbreviations

MRI: magnetic resonance imaging
mTOR: mammalian target of rapamycin
PET-CT: positron emission tomography-computed tomography
PMH: pseudomyogenic hemangioendothelioma
TSC1: tuberous sclerosis 1
